# Neuregulin 1 Type III/ErbB Signaling Is Crucial for Schwann Cell Colonization of Sympathetic Axons

**DOI:** 10.1371/journal.pone.0028692

**Published:** 2011-12-16

**Authors:** Stephan Heermann, Julia Schmücker, Ursula Hinz, Michael Rickmann, Tilmann Unterbarnscheidt, Markus H. Schwab, Kerstin Krieglstein

**Affiliations:** 1 Department of Neuroanatomy, University of Heidelberg, Heidelberg, Germany; 2 Department of Neuroanatomy, University of Göttingen, Göttingen, Germany; 3 Department of Neurogenetics, Max Planck Institute of Experimental Medicine, Göttingen, Germany; 4 Department of Molecular Embryology, Institute of Anatomy and Cell Biology, University of Freiburg, Freiburg, Germany; 5 FRIAS, University of Freiburg, Freiburg, Germany; The University of Akron, United States of America

## Abstract

Analysis of Schwann cell (SC) development has been hampered by the lack of growing axons in many commonly used *in vitro* assays. As a consequence, the molecular signals and cellular dynamics of SC development along peripheral axons are still only poorly understood. Here we use a superior cervical ganglion (SCG) explant assay, in which axons elongate after treatment with nerve growth factor (NGF). Migration as well as proliferation and apoptosis of endogenous SCG-derived SCs along sympathetic axons were studied in these cultures using pharmacological interference and time-lapse imaging. Inhibition of ErbB receptor tyrosine kinases leads to reduced SC proliferation, increased apoptosis and thereby severely interfered with SC migration to distal axonal sections and colonization of axons. Furthermore we demonstrate that SC colonization of axons is also strongly impaired in a specific null mutant of an ErbB receptor ligand, Neuregulin 1 (NRG1) type III. Taken together, using a novel SC development assay, we demonstrate that NRG1 type III serves as a critical axonal signal for glial ErbB receptors that drives SC development along sympathetic axons.

## Introduction

Schwann cells (SC), the main glial cell type of the peripheral nervous system, are derivatives of the neural crest, a transient structure emerging from the dorsal neural tube [1], [2]. Normal SC function is essential for the development and long term integrity of peripheral nerves. The number of SCs in peripheral nerves is adjusted to the number of axons, regulated by proliferation as well as apoptosis of SC precursors. Several factors have been identified that influence proliferation and survival of SCs as well as SC myelination, including TGF-beta [3] and Neuregulin (NRG) 1 [4], [5], [6], [7]. In contrast however, the molecular signals that control SC precursor migration along nerve fibers are not well understood. It is presumed that SC precursors originate from a pool of migrating neural crest cells which move to nerve trunks of developing efferent and afferent fibers and migrate along these fibers to ensheath the nerve [8]. NRG1 might influence SC migration, as demonstrated for rat SCs [9], [10] and also for a conditionally immortalized SC precursor cell line [11]. Genetic screens in zebrafish have revealed that ErbB2 and ErbB3, which serve as glial tyrosine kinase receptors for NRG1 in the PNS [12] are essential for SC migration along the zebrafish lateral line organ [13]. However, the molecular processes regulating development in zebrafish may not fully recapitulate those in mammals and analysis of SC migration in mammals is hampered by the inaccessibility of peripheral nervous tissues, such as the sciatic nerve or the sympathetic ganglia for time-lapse imaging in vivo. As a consequence artificial *in vitro* assays such as the “Scratch Assay” or the “Boyden Assay” are frequently used to address SC migration during development. Although these systems provide the opportunity to obtain quantitative data, they miss two important features. First, they lack axons the substrate along which SC migrate physiologically, and second, they normally do not address proliferation and apoptosis. In this investigation we took advantage of the ganglion explantation technique [14]. Only few studies have used a similar approach to analyze SC development [9], [15]. Using growing axons from explanted SCGs in combination with time-lapse imaging we studied the molecular processes involved in SC migration, proliferation and cell death along developing sympathetic axons.

## Materials and Methods

### Ethics Statement

All animal work was carried out in agreement with the local ethical committees. The University of Heidelberg/Regierungspräsidium Karlsruhe Referat 35 has approved this study (ID: T-07/10 and T-59/08).”

### Collagen gel preparation

Collagen gels were prepared according to a protocol of T. Ebendal [16], with slight modifications. Briefly, 455 µl of 10× MEM (Gibco), 112 µl of NaHCO_3_ (7.5%) (Gibco) and 50 µl of glutamine (200 mM) (Gibco) and 383 µl of 0.15- 1 M NaOH (Roth) were prepared as a concentrated medium. 210 µl of this concentrated medium were gently mixed with 800 µl of a dialyzed collagen stock solution, prepared from rat-tails. 50 µl of this mixture were applied to cell culture wells (96 well plates, Nunc) and maintained in cell culture incubator (37°C, 5% CO_2_ and humid conditions) until a solid matrix was assembled.

### Mice and tissue preparation

Time pregnancy matings of NMRI and s100 GFP mice were performed overnight with the day of the vaginal plug in the morning considered as day 0.5. At embryonic day 16.5 (E16.5) or 18.5 (E18.5) respectively, mothers were sacrificed by cervical dislocation and the embryos were harvested by cesarean section. In addition, time pregnant mice were ordered from Charles River (matings performed over day). NRG1 type III heterozygous mice [17] were crossbred for 3 nights. Embryos were harvested for SCG-dissection between E16.5 and E18.5 according to the same protocol as the NMRI mice. Genotyping of genomic DNA was performed using Chr8 sense Primer: 5′-ACTTTCTTCTTCCCATTCTGT -3′, Chr8 antisense Primer: 5′-TTTCTCTTGATTCCCACTTTG -3′ and NEO antisense Primer: 5′-TTTACTCTTCCTTACGGTCTA -3′.

Superior cervical ganglia (SCGs) of the embryos were dissected, consecutively cleaned in DPBS (Gibco) and placed on collagen gels.

### Cell culture experiments and treatments

SCGs on collagen gels were kept in serum free Neurobasal cell culture medium containing glutamine (2 mM), B27 (1×) and PSN (penicillin/streptomycin/neomycin) (1×) under humid conditions in 37°C and 5% of CO_2_. SCG explants of E16.5 and E18.5 embryos showed the same characteristics under culture conditions. All experiments were carried out under presence of nerve growth factor (NGF, R&D, stock in PBS with 0.1% BSA: 50 ng/µl, working concentration: 30 ng/ml) to facilitate optimal nerve growth from the SCG explants into the collagen gel matrix. In addition to NGF, EGFR/ErbB-2/ErbB-4 inhibitor (ErbB inhibitor/ErbB-inh)(Calbiochem, stock in DMSO: 4 µg/µl, working concentration: 0.8 ng/ml) was used to inhibit ErbB signaling at the level of the receptor (ErbB2/4). Experiments with the ErbB inhibitor were carried out in different variations. In the first variant the inhibitor was added at DIV (Day *in vitro*) 0 and the experiment duration was either until DIV5 or until DIV8, in the latter case with change of medium at DIV4. In the second variant the inhibitor was added at DIV4/5 and the duration of the experiments were until DIV9/10. For the third variant the inhibitor was added at DIV3 with the experiment being terminated at DIV4. In an additional set of experiments an inhibitor of apoptosis, a caspase inhibitor (casp-inh, Caspase inhibitor Z-VAD-FMK, Promega, 20 mM stock) was co-applied with the ErbB-inhibitor at a concentration of 40 µm. Where indicated Bromodeoxyuridin (BRDU 1∶500, Fluka/Sigma) was added to the medium for the last 6 hours of the experiment.

Experiments with Neuregulin 1 type III deficient SCGs were performed for 6 days with time lapse imaging throughout the whole period.

### Time-lapse imaging and supplementary movie files

Where stated, SCG explants were imaged in near live time. The time-lapse movies were recorded with the following frame rates. Movie S1 with 10 minutes, movie S2 with 30 minutes, movies S3 and S4 with 10 minutes, movies S5, S6, S7, S8, S9, S10 with 10 minutes and movies S11 and S12 with 20 minutes. Files were assembled with jepg compression via ImageJ software (scale bars = 100 µm). Time-lapse imaging was performed with a Leica time-lapse imaging setup LASAF6000 and a Nikon time-lapse imaging setup (Nikon Imaging Center Heidelberg). The tissue was meanwhile incubated under humid conditions, 5% of CO_2_ and 37°C.

### Image analyzes, quantification and statistical evaluation

For quantitative analyses of distances from the last SC to the axonal tip and the proliferation index after ErbB-inh treatment (BRDU/DAPI) the explants were cryosectioned and sampled. For measuring distances 18 control and 18 ErbB-inh treated SCG explants were used for estimation of the proliferation index 19 control and 18 ErbB-inh treated explants. For quantification of the distance from the SCG explant to the furthest migrated SC and the axonal length, explants were processed as whole mounts. To facilitate optimal conditions for image acquisition with subsequent measurements, the whole mount SCG explants were dried on microscope slides. Distances and lengths were measured from the explants to the periphery. For quantification of migration distances in the case of ErbB-inh/Casp-inh cotreatment 21 control and 20 inhibitor treated ganglia were analyzed. For quantification of Tunel positive vesicles after ErbB-inh/Casp-inh cotreatment 4 control and 5 treated ganglia were analyzed.

For quantification of migration distances after ErbB-inh treatments, 23 inhibitor treated and 22 control explants were used, for quantification of the axonal length 12 control and 12 ErbB-inh treated explants. Migration distances after aphidicolin were quantified by using 12 control and 12 aphidicolin treated explants. For quantification of apoptosis, 9 control and 8 ErbB-inh treated explants were used and for quantification of proliferation after aphidicolin treatment before imaging, 4 control and 8 aphidicolin treated explants. For both latter analyses whole mounts were sampled. Distances and cell counts were performed software based, using the NIH software ImageJ. Separate SCG explants were taken as separate experiments for statistical evaluation. Statistical data analyzes were performed by t-tests using Graph Prism software (Graphpad.com).

### Immunohistochemical analyzes

Explant containing collagen gels were fixed with 4% PFA at the end of an experiment, where indicated, and either further cryoprotected in 30% sucrose solution over night with consecutive free floating cryosectioning (80–120 µm thickness freezing microtome) (Reichardt- Jung) or directly taken for whole mount processing.

Immunohistochemistry, using standard protocols for antibodies against TH (Chemicon, rabbit, polyclonal, 1∶1000–2000, mouse anti-TH, 1∶400) S100 (Sigma, mouse, monoclonal, 1∶100) pHH3 (Milipore, rabbit polyclonal, 1∶ 400) was performed. Briefly, sections were blocked in PBS containing 10% normal donkey serum and 2% Triton ×100 for 2 hours with consecutive antibody incubation in blocking solution over night. The next day, after washing, the sections were incubated with labeled secondary antibodies (donkey anti rabbit/donkey anti mouse/FITC/Alexa488/CY3). Further, sections were mounted on gelatine coated microscope slides and air-dried. In the end nuclear counterstaining (DAPI, 1∶10000) was performed for 5 minutes with consecutive washing and mounting in aqueous mounting medium (DAKO/Mowiol). Explants used for whole mount immunohistochemistry were dried on microscope slides, for best analyses in two dimensions. Images were taken by either conventional fluorescence microscopy with Zeiss and Olympus microscopes or with confocal microscopy with a Leica confocal SPE. BRDU Immunohistochemistry was performed with an antibody (Abcam sheep polyclonal 1∶100) according to the manufacturers recommendations with slight variations. Briefly, sections were incubated in 1N HCL 10 min on ice, in 2N HCL 10 min at room temperature and 10 min at 37°C followed by incubation in 150 mM borate buffer (pH 8,4). Blocking was performed with 10% NDS (Normal Donkey Serum) and 2% Triton X-100 in PBS for 1.5 hours. The following steps were according to standard immunohistochemistry, mentioned above. Tunel stainings were performed according to manufacturers description (Dead End Fluorometric Tunel System, Promega) with slight adjustments owing to whole mount stainings.

### RT-PCR

For RT-PCR, SCG tissue, explanted at embryonic day 16, harvested at DIV3 was taken. RNA was isolated according to a standard protocol and reversely transcribed. PCR for Neuregulin 1 subtypes was performed with the following forward primer, for Neuregulin 1 type I: GCCGAAGGCGACCCGAGC, for Neuregulin 1 type II: ACAGCAGGTACATCTTCTTCATGGA and for Neuregulin 1 type III: TCAGGAACTCAGCCACAAACAACAG, combined with the reverse primer: TTTTGCAGTACGCCACCACACACAT respectively. Adult wildtype cortex (ctx) was used as positive control.

## Results

### Time lapse imaging of Schwann cell migration along sympathetic axons

**Figure 1:**
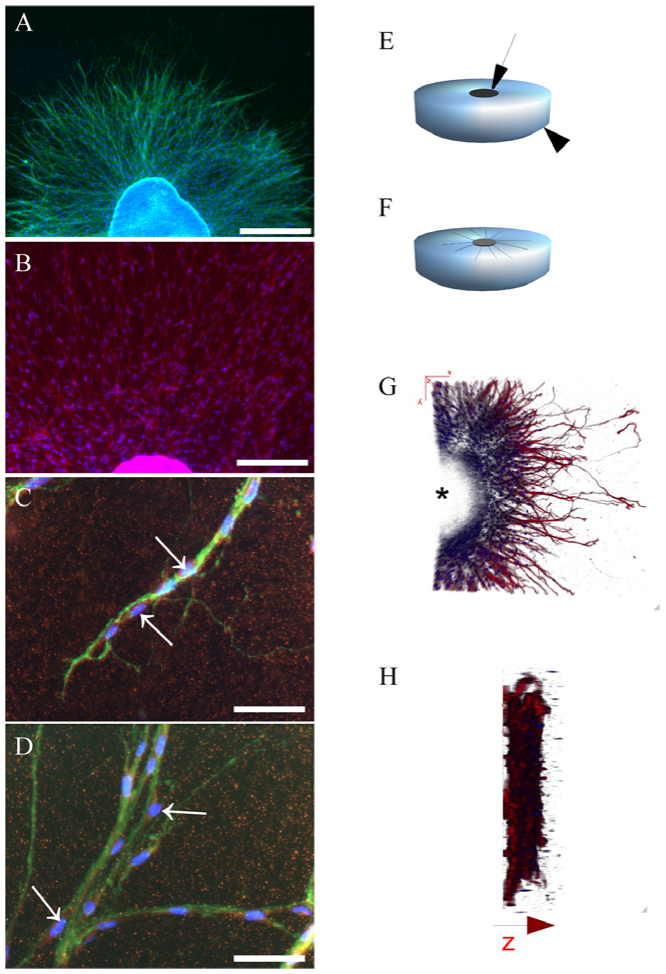
Schwann Cell Migration Assay using NGF induced axon outgrowth from SCG explants. SCG explants derived from mouse E16–E18 were cultured on collagen gels and treated with NGF (30 ng/ml). A: Overview, immunohistochemistry for TH (green) with DAPI nuclear counterstaining, explant at DIV4 (scalebar = 500 µm). B: Overview, immunohistochemistry for S100 (red) explant at DIV4 (scalebar = 200 µm). C/D: Immunohistochemistry for TH (green) with nuclear counterstaining DAPI (blue). Clear (red) S100 positive cells are attached to the TH positive axons (arrows). (scalebar = 50 µm) Note that after sectioning and performance of immunohistochemistry, Schwann cells can also be found close to the axonal endings.

To analyze SC migration, explants of embryonic (E16–18.5) mouse superior cervical ganglia (SCGs) were grown on a collagen matrix (Fig. 1E). In the presence of NGF (30 ng/ml), axons elongate from the SCG explants into the matrix (Fig. 1A, F, G, H), followed by a wave of migrating cells from the ganglion towards the periphery (Fig. 1B, movie S1). Penetration of axons into the matrix can be appreciated in the 3D reconstruction performed with a confocal z-stack scan (Fig. 1 G, H, movie S13). For time lapse imaging also a transgenic mouse line was used in which SCs and SC precursors were marked by GFP expression under control of the human S100 promoter [18] (movie S1 and S6). Furthermore, by immunostaining for S100, migrating cells were identified as S100-positive SCs (Fig. 1 B, C and D). Thus, this assay has two major advantages. First, it models in vivo SC migration along outgrowing axons. Second, it is accessible to time lapse imaging (movie S1, S2, S5, S6). These features demonstrate the experimental power of the SCG explant assay compared to other SC migration assays frequently used in mouse or other rodents.

### ErbB inhibition prevents colonization of distal axonal compartments

**Figure 2:**
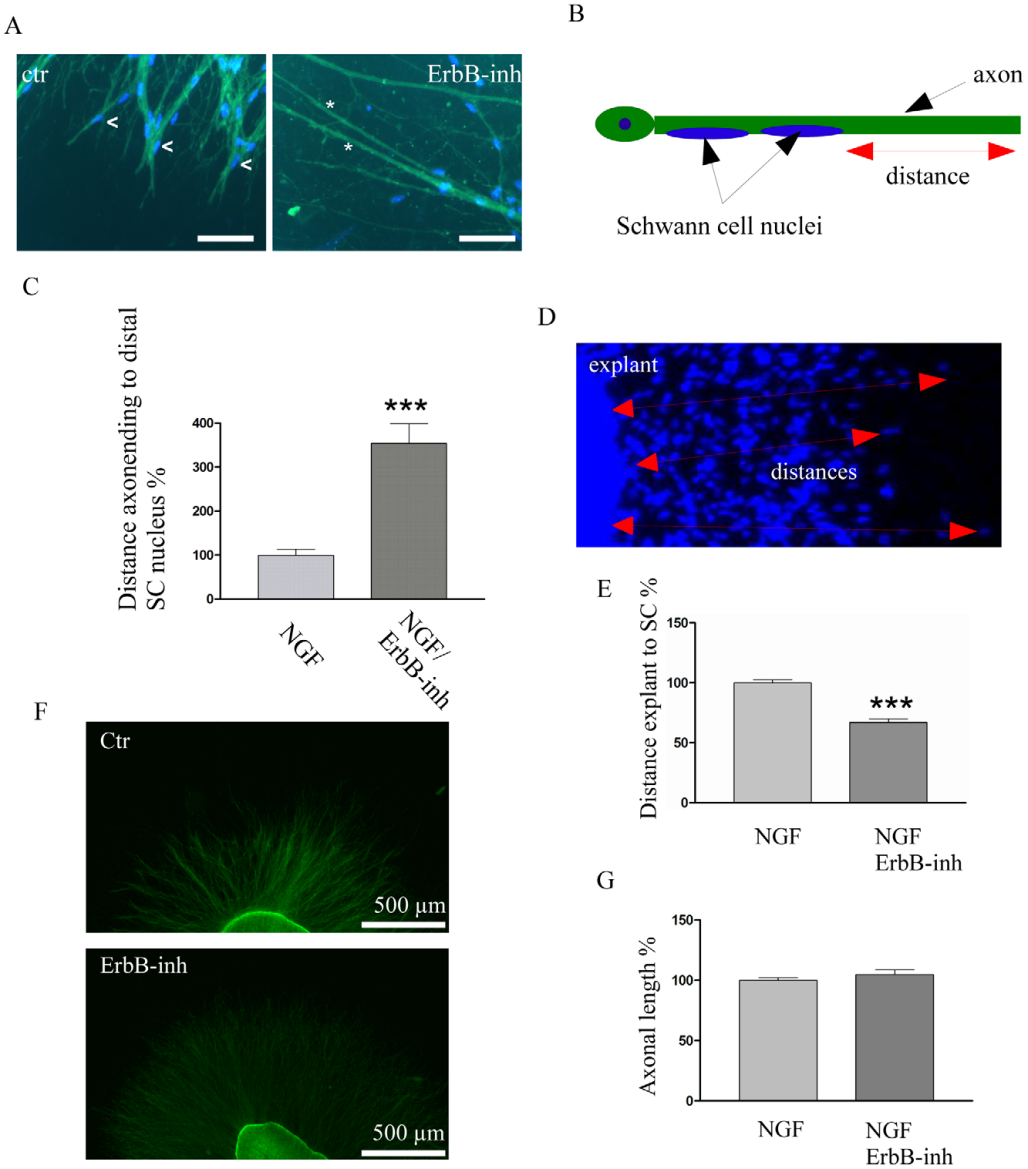
ErbB signaling is important for Schwann cell migration. A: Immunohistochemistry (DIV9/10), TH (green) and DAPI (blue) of an NGF treated control (ctr) and and NGF/ErbB-inh treated (starting DIV4/5) sample (scale bars = 50 µm). Ctr: NGF treated SCG explant, ErbB-inh: combined NGF and ErbB-inh treated SCG explant (E16.5). Note the presence of SC nuclei in the proximity to the axonal endings (arrowheads) in the control, whereas in the ErbB-inh treated probe large uncovered areas are visible (asterisks). B: scheme showing the first method for quantification of the distance between the distal Schwann cell nucleus and the axonal tip. C: Quantification of the distances between the distal SC nucleus and the axonal tip. Quantification has been performed for inhibitor treatments starting at DIV4/5 (t-test: p value<0,0001). Distances are given in percentage to the respective NGF treated controls which are set as 100%. Graphs are presented as mean and +/− SEM. Note the drastic increase of the distances. D: Scheme showing the second method for quantification of distances between SCG explants and SC nuclei treated from DIV3 to DIV4 on. E: Quantification of mean distances between SCG explant and the furthest SC nucleus. (t-test: p value<0,0001). The solely NGF treated controls were set to 100%. Graphs are presented as mean and +/− SEM. F: TH immunohistochemistry (green) of a control and a ErbB-inh treated sample. G: Quantification of axonal lengths between ctr and ErbB-inh treated samples. The mean axonal length of the control was set to 100%. (t-test: p value = 0.3105) Graphs are presented as mean and +/− SEM.

ErbB signaling was shown to be active during SC development in the somatic peripheral nervous system [19]. Thus, we wanted to address whether ErbB signaling is also relevant for SC development in the sympathetic nervous system. To this end, we blocked ErbB signaling with a pan-specific ErbB inhibitor (ErbB-inh,) [20]. Numerous SCs can be seen migrating along axons in solely NGF-treated SCG explants (movie S3). In contrast, only bare axons are visible when explants are additionally treated with the ErbB-inh starting at DIV0 (movie S4). Interestingly, when kept in culture for a long period (until DIV5/DIV8) SCs can be found again migrating along axons. This indicates that the ErbB-inh when added at DIV0 is not killing the entire pool of SC precursors within the ganglion. To analyze whether inhibition of ErbB signaling can block the migration of SCs that have already colonized sympathetic nerves, ErbB-inh treatment was started with a delay following a pretreatment with NGF only. Traveling distances of SCs were quantified by measuring two parameters. One parameter was the mean distance between the most distally located axon associated SC nucleus and the tip of the axon (scheme, Fig. 2B). The second parameter was the mean distance from the SCG explant to the most distally localized SC (scheme, Fig. 2D), which reflects the distance SCs have migrated. The distance from the most distally localized SC nucleus to the tip of the axon is increased dramatically after ErbB-inh treatment (3.5 fold) compared to the controls. Also SC nucleus-free axonal regions were seen after Erb-inh treatment, ranging from 130–370 µm. To determine migration distances, SCG explants were treated with ErbB-inh at DIV3 until the assay was terminated at DIV4. The mean distance of the furthest SC nucleus to the explant (Fig. 2D and E) was measured. Importantly, after ErbB-inh treatment the mean distance from the margin of the explant to the most distal SC (Fig. 2D/E) was reduced to 70%, compared to controls (Fig. 2E). These data strongly suggest that blocking ErbB receptors slows SC colonization of distal axonal compartments. Importantly, axonal length might be influenced by SCs or itself influence the distances between the leading SCs, the explant and the axonal tip. To this end, TH-ir positive axons were measured from the border of explants to the periphery at DIV4 after treatment with the ErbB-inh at DIV3. When compared to controls only a very mild increase in axonal length was observed (Fig. 2F/G).

### ErbB signaling promotes SC proliferation

**Figure 3:**
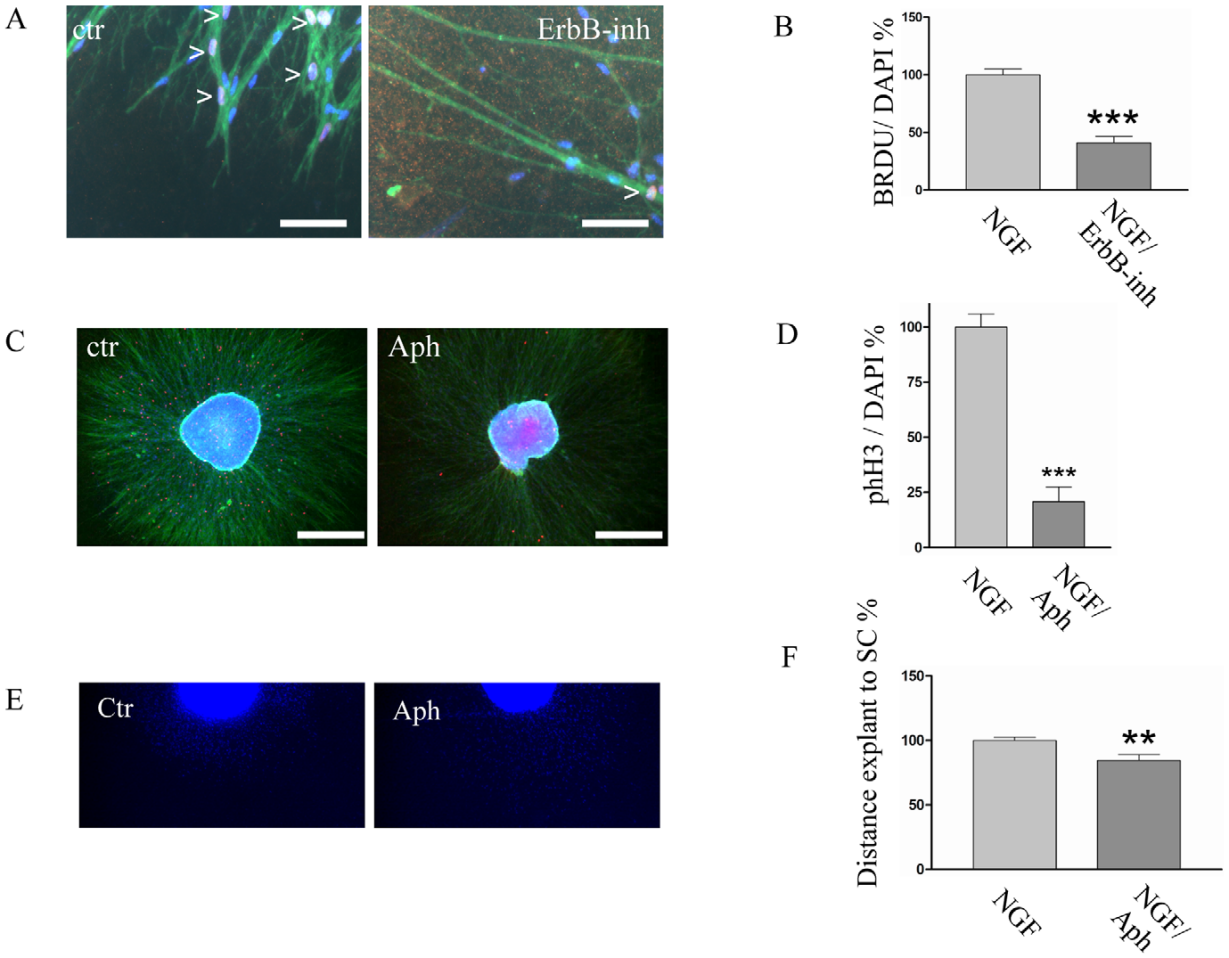
ErbB signaling is affecting Schwann cell proliferation. A: delayed treatment of SCG explants (starting at DIV4/5) and additionally analyses of BRDU incorporation. Immunohistochemistry, TH (green), BRDU (red) and DAPI (blue) of control (ctr) and ErbB-inh treated (ErbB-inh) samples. Whereas in the control many nuclei (DAPI/blue) are also positive for BRDU (red) (arrowheads), in the ErbB-inhibitor treated sample there are only very few nuclei positive for BRDU (arrowhead). B: Analysis of the DAPI/BRDU ratio. After treatment with ErbB-inh SC proliferation is decreased drastically. T-test: p-value = 0,0001. Graphs are presented as mean and +/− SEM. C: Immunohistochemistry TH (green), phH3 (red) and DAPI (blue). Control (ctr) Aphidicolin (Aph), note the difference in the number of phH3 positive cells. D: Analysis of the proliferation index (pHH3/DAPI). B: Quantification of proliferation index (pHH3/DAPI) (t-test: p value<0,0001) Graphs are presented as mean and +/− SEM. E: DAPI nuclear staining of a control (ctr) and a Aphidicolin (Aph) treated sample. F: Quantitative analyses of migration distances between control and Aphidicolin treated sample. (t-test: p value = 0.0062) Graphs are presented as mean and +/− SEM. Note that a mild reduction of the migration distance is visible after Aphidicolin treatment.

Since proliferation and survival of SCs may affect their migraton, we asked whether ErbB signaling affects SC mitosis. Thus, we employed a delayed treatment of SCG explants (DIV4/5 until DIV9/10) with ErbB-inh and added BRDU to the culture medium for the last 6 hours of the experiment. For quantitative analysis the explants were cut and immunostained for TH and BRDU combined with a DAPI nuclear counterstaining (Fig. 3A). When we determined the BRDU/DAPI proliferation ratio (Fig. 3B) we observed a reduction to less than 50% in the ErbB-inh treated explants. To address whether proliferation and migration functionally interact, we aimed at uncoupling both processes. To this end we used Aphidicolin, a cytostatic drug, which retains cells in the early S phase of mitosis by blocking DNA polymerases [21]. The impact of Aphidicolin on SC proliferation was directly validated by immunostaining for phosphorylated histone 3 (pHH3), a marker for cell mitosis. Quantification showed a massive reduction of pHH3-positive axon associated SCs (Fig. 3 C/D). We want to note that even though Aphidicolin acts as a cytostatic drug we observed dead SC nuclei, especially after a longer treatment over two days. ƒs S7/S7a show explants treated with Aphidicolin. Throughout imaging SC migrating could be observed, in Aphidicolin treated explants (movie S5/S6), whereas directed SC migration was halted after a treatment with ErbB-inh (movie S7/S8, see movies S5 and S6 as controls). However, although SC migration was not blocked by Aphidicolin treatment (movie S9/S10), migration distance was modestly reduced (Fig. 3 E/F).

### ErbB signaling affects SC migration indirectly

**Figure 4:**
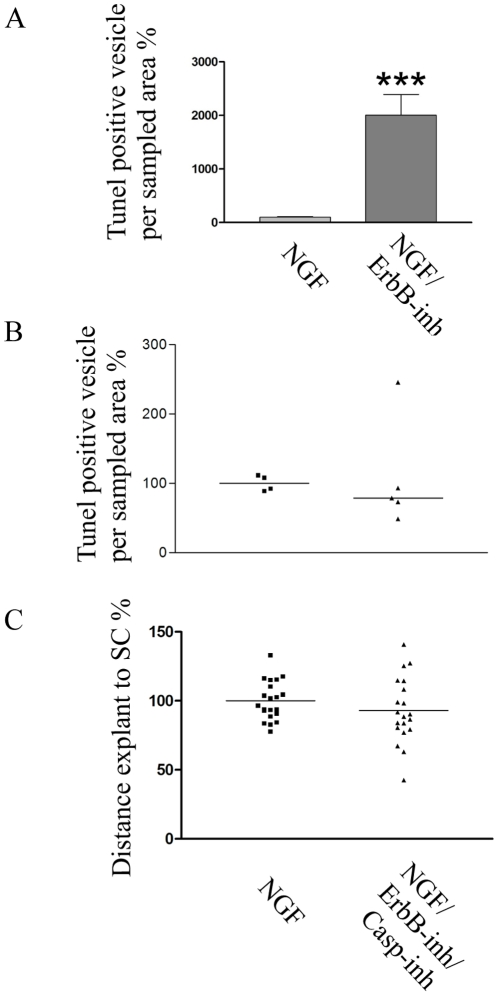
ErbB inhibition interferes with SC migration indirectly. Quantitative analyses of Tunel positive vesicles of NGF treated control versus ErbB-inh treated samples (t-test: p value<0.0001). Graphs are presented as mean and +/− SEM. A strong increase in Tunel positive vesicles is visible in the ErbB inhibited explants. B: Quantitative analyses of Tunel positive vesicles after a combined treatment with ErbB-inh and Casp-inh (t-test: p value = 0.8553). Graphs are presented as scatter blot with mean. Note that the Casp-inh potently inhibits apoptosis. C: Quantitative analyses of migration distances after a combined treatment with ErbB-inh and Casp-inh (t-test: p value = 0,2706). Graphs are presented as scatter blot with mean to also show the inner group variance.

To address the effect of ErbB inhibition on SC apoptosis, explants were analyzed by Tunel staining and quantification of apoptotic vesicles in sampled areas. A dramatic increase of apoptotic vesicles in ErbB-inh treated samples compared to controls (Fig. 4A) was observed. To investigate whether increased cell death affects the migratory potential of the surviving SC pool, we employed a combined delayed treatment with the ErbB-inh and a Caspase inhibitor (Casp-inh) to block apoptosis. The anti apoptotic activity of the Casp-inh was estimated by Tunel staining and quantification of apoptotic vesicles in sampled areas in comparison to control explants. The Casp-inh applied in combination with the ErbB-inh significantly reduced apoptosis (Fig. 4B) to levels comparable to controls. Surprisingly however, after blocking both ErbB signaling and apoptosis, no difference in migration distances could be observed (Fig. 4C) when compared to control explants. This suggests an indirect negative effect of ErbB inhibition on SC migration and colonization of distal axonal compartments.

### The type III variant of NRG1 is crucial for SC colonization

**Figure 5:**
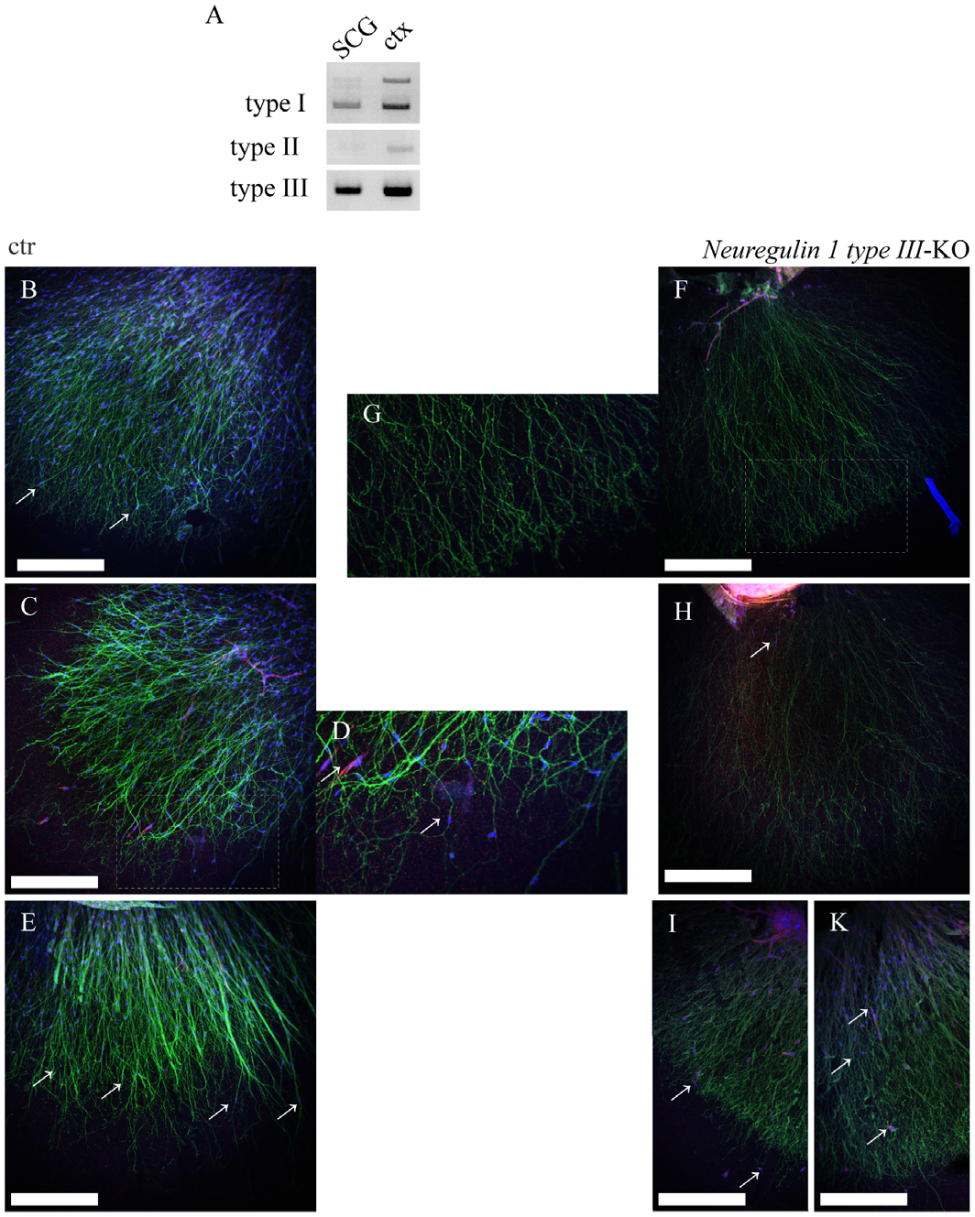
Analyzes of Neuregulin 1 type III deficient SCG explants. A: RT-PCR for Neuregulin 1 splice variants from SCG RNA. Tissue was explanted at E16.5 and harvested at DIV3. Adult wildtype cortex was taken as positive control. Note that Neuregulin 1 type III is strongly expressed, whereas type I is only weak and type II is almost not expressed. B–K: Confocal images (scalebar 100 µm) B–E: Immunostainings of control explants, TH (green), s100 (red) with DAPI nuclear counterstaining (blue), D: close up of boxed area in C, nuerous SCs can be seen in proximity to axonal tips (arrows). F–K: Immunostainings of Nrg1 typeIII- KO explants, TH (green), s100 (red) with DAPI nuclear counterstaining (blue), G: close up of boxed area in F, SCs can be seen almost exclusively in proximal regions (F, G and H) with only few exceptions (arrows in L and M).

Multiple NRG1 isoforms are produced by alternative splicing and grouped into three subclasses according to the presence of distinct N- terminal domains [22]. We examined which of these isoforms may act upstream of glial ErbB receptors during SC migration in the sympathetic nervous system. To identify NRG1 isoforms expressed in SCGs during development, RT-PCR was performed on SCG RNA prepared at DIV3 from SCGs explanted at E16.5. SCGs displayed prominent NRG1 type III expression, whereas type I was only weakly expressed and type II virtually absent (Fig. 5A). We therefore analyzed SC migration in SCG explants derived from NRG1 type III deficient mice and control littermates (E16.5–18.5). In NRG1 type III-deficient SCG explants normal axonal outgrowth was detected (Fig. 5 B–E, movie S12, ctr: movie S11).

In contrast, we observed a strongly reduced number of migrating SCs along elongating axons of NRG1 mutants when compared with control explants (Fig. 5 B–E). As a result we observed SCs in proximal regions (Fig. 5 F–K), but distal regions of NRG1 type III mutant axons almost completely lacked SCs (Fig. 5 F–H), with only a few exceptions (Fig. 5 I/K). We conclude that axonal NRG1 type III is required for the SC colonization of distal compartments of sympathetic axons.

## Discussion

A more detailed understanding of the molecular mechanisms that regulate SC development has been hampered by methodological restrictions. Many studies to date used “Boyden assays” or “Scratch assays” to analyze SC migration. Here, we employed a SC migration assay, which has been adapted from the classical ganglion explant technique [14]. This assay more closely mimics development in vivo, as it contains axons the physiological substrates for SC migration and allows SC migration out of an explanted SCG along outgrowing axons to the periphery. Only few others have used similar approaches [9], [15].

We identify signaling by ErbB receptor tyrosine kinases as a major regulator of SC development in the sympathetic nervous system with respect to SC proliferation, survival and colonization of distal axonal areas. Pharmacological blockade of ErbB2/4 potently reduced SC proliferation and induced apoptosis. Furthermore, SC colonization of distal axonal compartments was severely affected by ErbB inhibition as demonstrated by time-lapse imaging as well as by quantification of migration distances.

To address the different phenotypes observed and to investigate how these phenotypes interact, we employed several assays, in which we pharmacologically uncoupled ErbB signaling from SC proliferation and survival. The massive SC migration out of an explant (movies S1/S2) indicates that proliferation is necessary to provide the large number of migrating cells. However, since inhibition of SC proliferation (without blocking ErbB signaling) did not prevent migration (movies S9/S10) and only mildly affected the colonization of distal axonal compartments in the investigated time frame, we propose that SCs can migrate along sympathetic axons in the absence of cell division, similar to findings in the peripheral nervous system of zebrafish [13]. Furthermore, we investigated the interplay between SC apoptosis and migration. Unexpectedly, while the inhibition of ErbB signaling severely affected the migration of SCs to distal parts of sympathetic axons (Fig. 2), normal SC migration was restored when apoptosis was blocked together with ErbB signaling. This finding argues against an essential direct role for ErbB signaling in the migration of SCs along sympathetic axons and is at odds to studies in zebrafish [13]. However, since previous studies have shown that much lower levels of ErbB signaling are required to promote SC migration compared to proliferation [10], we cannot exclude the possibility that residual ErbB activity persists in our explant assay after pharmacological treatment, which might be sufficient to restore migration when massive SC apoptosis is blocked. Also other factors could compensate when ErbB signaling is blocked but Schwann cell survival is maintained. Potential candidates for this, also shown to influence SC motility, are GDNF [11] NGF [23] and IGF-1 [24].

Finally, we provide data, which suggest the splice variant NRG1 type III, as the major axonal ligand for ErbB receptors to promote SC colonization of sympathetic axons. Meyer et al [25] suggested this splice variant to be expressed in sympathetic ganglia at E12. Here we show that NRG1 type III continuous to be expressed at a late embryonic stage. Migration assays using NRG1 type III-deficient SCG suggested an essential role of NRG1 type III for the directed migration of SCs along sympathetic nerves. Since a reduced number of SCs persists in the proximity of explanted NRG1 type III-deficient sympathetic ganglia, we argue that impaired migration cannot be explained solely by the loss of (premigratory) SCs. Findings in NRG1 type III null mutants from a previous study [17] also demonstrated a lack of SCs in distal areas of peripheral nerves. Reduced SC numbers in distal axonal areas were explained by impaired proliferation and increased apoptosis of SCs in the absence of NRG1 signaling, which acts as a SC mitogen and survival factor in vitro [4], [26], [27].

However, this interpretation does not fully explain, why in our study SCs are present in the vicinity of explanted ganglia but fail to colonize distal areas. Considering our data regarding ErbB receptor blockade, it is conceivable that reduced survival in the proximal regions of axons leads to stalling or even backward movement of viable migrating SCs, which would indirectly impair the colonization of distal axonal compartments. Aberrant migration (including ‘backward’ movement) has been observed after pharmacological blockade of ErbB signaling in zebrafish [13]. A reason for not observing large amounts of dead SCs along NRG1 typeIII deficient axons as we did after ErbB blockade could be due to the experimental setup. Interfering with ErbB signaling of migrating wildtype SCs was performed at DIV3 and led to massive SC death. In the other experimental setup, NRG1 typeIII was genetically depleted and absent from the start of the experiment. Therefore a steady state could have been adjusted earlier. In addition, ErbB inhibition led to reduced proliferation. Although SCs were able to migrate in general when cell division was blocked, a reduced proliferation from the beginning of the experiment could lead to a massively reduced number of SCs. Finally we propose that due to reduced proliferation and survival the few SC in the vicinity of NRG1 type III KO SCG explants rather stall than migrate to distal compartments.

In summary the SCG explant culture is a powerful tool to investigate SC development. We used this assay and characterized the role of NRG1/ErbB signaling for SC proliferation, apoptosis and colonization of distal axon compartements during peripheral sympathetic axon development.

## Supporting Information

Movie S1 S100-GFP (E17.5), imaging start DIV2, until DIV3.
(AVI)

Movie S2 NGF treated (E18.5), imaging start DIV1, imaging duration 2 days.
(AVI)

Movie S3 Control to ErbB-inh (E16.5) movie 4, imaging start DIV2, imaging duration 2 days.
(AVI)

Movie S4 ErbB-inh (E16.5), imaging start DIV2, imaging duration 2 days (factors added DIV0).
(AVI)

Movie S5 Ctr (E17) to movies 7 and 9, imaging DIV3–DIV4.
(AVI)

Movie S6 (E17.5) ctr to movies 8 and 10, imaging DIV3–DIV4, arrows show direction of SC migration.
(AVI)

Movie S7 ErbB-inh (E17), imaging DIV3–DIV4 (factors added at DIV3).
(AVI)

Movie S8 (E17.5) ErbB-inh, imaging DIV3–DIV4 (factors added at DIV3), arrows show direction of SC migration.
(AVI)

Movie S9 Aphidicolin (E17), imaging DIV3–DIV4 (factors added at DIV3).
(AVI)

Movie S10 (E17.5) Aphidicolin, imaging DIV3–DIV4 (factors added at DIV3), arrows show direction of SC migration.
(AVI)

Movie S11 *Neuregulin 1 type 3*-ctr (E16–18.5) to movie 12, imaging start DIV0, duration for 6 days.
(AVI)

Movie S12 *Neuregulin 1 type 3*-KO (E16–18.5), imaging start DIV0, duration for 6 days.
(AVI)

Movie S13 3D reconstruction of a confocal z-stack of an control explant.
(AVI)
